# Supplementary material for: Parasitic nematode fatty acid- and retinol-binding proteins compromise host immunity by interfering with host lipid signaling pathways
Source: PLoS Pathog. 2021 Oct 29;17(10):e1010027. doi: 10.1371/journal.ppat.1010027 (PMC8580252; doi:10.1371/journal.ppat.1010027)
Supplement: Attachment — Submitted filename: Reviews of Parks comments_Reviewedv3.pdf [file ppat.1010027.s017.pdf]

Response to Reviews of Parks et al., 2021

Editor:
While the reviewers found this work intriguing, there is a concern about the in vivo physiological relevance of the over expressions data. Please address the issues raised by the reviewers in Figures 1 and 4 as well as address the fatty acid modulation induced by natural Sc infection.

We thank the editor for taking the time to edit our manuscript and oversee the review process. We have thoroughly considered the reviewers' concerns and have responded to each as outlined below. We were able to address their concerns with the following additional experiments: 1) nematode infections of adult flies, 2) a FAR-1 and FAR-2 co-injection to identify any additive effects, 3) overexpression of an irrelevant protein in transgenic flies to further validate our FAR-overexpression data, 4) and a new hemolymph lipidomic analysis of FAR-2-expressing flies. These four additional experiments round out our findings that are the first to show that nematode FARs are detrimental to the outcome of infection and are directly immunomodulatory in an animal system. We have carefully discussed the *in vivo* physiological relevance of the data we have presented. We have also addressed the issues raised regarding Figures 1 and 4, and we have addressed the concern regarding fatty acid modulation induced by natural Sc infection.

Reviewer 1:
Overall, the authors have meticulously shown the effect of Sc-FARs in immunomodulating the host immunity during infection and specifically in this paper by modulating the lipid signaling molecules.

No major issues were identified.

<u>Minor Issues:</u>
I wonder if the authors have investigated whether the diminished Melanization, phenoloxidase and antimicrobial peptide activity is due to the effect of the FAR proteins on the Toll, Imd and/or TGF-b pathways upon bacterial infections. It was shown that TGF-β signaling in D. melanogaster is also regulated after parasitic nematode infection (PMID: 31316388). A discussion of these aspects can expand on the mechanism(s) by which the FAR proteins are the most effective, and thus possibly support similar research focused on immunomodulation in other host-parasite systems.

We thank the reviewer for their review and this suggestion. We agree that this is an interesting question and one that we will ultimately pursue. It was interesting that both FAR-1 and FAR-2 had a significant effect on *Drosomycin* and *Defensin* expression, suggesting that it has an impact on both Toll and Imd pathways. This is worth further study, but we think it is beyond the scope of the current study. We plan to move in this direction as we continue our research.

Reviewer 2:
The authors provide evidence for an immunesuppressive role for nematode derived FARs in vivo using a fly model, by showing that recombinant FAR administration or ectopic expression of FARs in drosophila flies renders them less resistant to bacterial infection. This was associated with a reduction in several immune response readouts. They additionially show that FAR1 and FAR2 can bind several lipid mediators.
The main strength and novelty of this study is the use of an in vivo model to assess the in vivo portential of FARs to modulate host immune responses. However, it was already shown that

FARs bind fatty acids, that they can be immunemodulatory and therefore this study, while solidly executed, does bring reltively little new to the able. Moreover, the physiological relevance of the concentartions of FARs reached in vivio following injection or by expression in these flies remains unclear. Finally the relevance of the FAR binding to the FAs for their effect is unclear, but would be important to provide true novel mechanistic insight

We appreciate the reviewer's evaluation of the work presented here, but we are unaware of any previous study providing experimental data that FARs are immunomodulatory in any animal system. There are a few studies demonstrating this in plants, but our manuscript represents the first experimental evidence that FARs can modulate animal immunity. There are approximately 40 papers on FAR proteins from 1985 through 2020. ~15 of those are primary research papers on FAR proteins from animal-parasitic nematodes, all of them suggesting without direct experimental evidence that FARs are immunomodulatory effectors. There are ~13 primary research papers on FARs in plant-parasitic nematodes. One of them (Iberkleid et al., 2013) provides phenotypic *in vivo* data that plants overexpressing FAR are more susceptible to nematode infections. There are several other papers providing evidence of immune modulation of FARs in plants in the form of decreased transcription of plant immunity effectors. Because our manuscript is the first to provide experimental evidence supporting the long-held belief that FARs are immunomodulatory in animals, we suggest that the data presented here are novel and important in developing the field.

Iberkleid I, Vieira P, de Almeida Engler J, Firester K, Spiegel Y, et al. (2013) Fatty Acid-and Retinol-Binding Protein, Mj-FAR-1 Induces Tomato Host Susceptibility to Root-Knot Nematodes. PLoS ONE 8(5): e64586.

Major Issues:

1) the physiological relevance of the findings remains unclear. specificically what is the physiological relevance of 250ng FAR administration? along the same what is concentration is reached of FARs in vivo in genetically modified Dm flies? Is this similar to what is found during natural Sc infection? These questions should be addressed by determining FAR concentrations during a natural Sc infection and in flies genetiically modified to express these FARs.

We agree with the reviewer that physiological relevance is an important consideration in evaluating these experiments. Previous work has found that Sc IJs release an average of 0.06 ng of total protein, in a 3-hour period (Lu et al. 2017). We can extrapolate this to suggest that an infecting population of 100 IJs can release ~50 ng of total protein in a 24-hour period, assuming that the amount of release is constant over that period. Sc releases hundreds of proteins (~472) during active infection (Lu et al. 2017). The two FAR proteins that we are working with would represent a small portion of the total protein released by the nematodes; they are among the most abundant 140 proteins in the complex mixture of 472. Therefore, a 250 ng dose is above what is physiological. However, we find it striking that we can detect a potent, robust, immunomodulatory effect from a 20 ng dose of FAR-2 and a 50 ng dose of FAR-1. These doses are near physiological and have potent effects. These FAR proteins have evolved to work in concert with the other ~470 proteins released by these nematodes, many of which are thought to be immunomodulatory. We have taken a reductionist approach to try to understand the function of FAR proteins, isolating them from among the total protein released by Sc IJs during active infection. While we acknowledge that the concentrations we have used are above but near physiological concentrations, we were surprised to see any phenotypic effect, given that the fly immune system has evolved to be robust against parasitic attack.

We did mention physiological relevance in the discussion section, on page 15: “Although 250 ng is likely greater than physiological concentrations of FAR during EPN infections, it is remarkable that such a phenotype was observed with only a single component of a complex array of venom proteins found in ESPs, where hundreds of proteins usually act in concert to dampen host immunity.”

Regarding the concentration in the overexpression flies, we are not sure how much FAR is produced. However, we have shown that there is little to no effect on the fly health in terms of survival. Certainly, the amount of FAR in the overexpression model will be above physiological levels in an *in vivo* infection, but as mentioned above, in an *in vivo* infection, there are hundreds of additional proteins released by the nematodes. Overexpression is a reasonable way to understand the function of individual effectors.

Lu D, Macchietto M, Chang D, Barros MM, Baldwin J, Mortazavi A, et al. (2017) Activated entomopathogenic nematode infective juveniles release lethal venom proteins. PLoS Pathog 13(4): e1006302.

2) the changes in lipid composisition in vivo following FAR exposure are interesting. To establish whether this of physiological relevance the author should assess whether during a naturla Sc infection a similar change in lipid compostion is observed.

We appreciate this point and understand the reviewer’s concern. Although there is one paper in plants that shows FAR increases susceptibility to nematode infections (Iberkleid), there are two obstacles to addressing this as part of the present study. The first obstacle is that flies are not a good host for nematode infections. The flies are small and are likely never encountered by Sc in the wild. Sc’s reproductive success is related to the size of the host it infects, making the fly a poor host as it likely can only house a few adult Sc, and the nematodes may not have robust attraction to flies (Griffin, 2012). We did perform a set of experiments to address this concern, and that data is presented as S-Fig. 1. We found that all infected flies die quickly (<4 days). Flies that are not infected live normal lifespans. After performing Sc nematode infections at a 100 IJ/host dose, we found that once a fly was infected with even one nematode it was unable to recover from the infection. This makes it especially difficult to determine variation in immune responses since the infection is rapidly lethal at any dose. As with bacterial infections, various fly strains sustain infections differently. We find no evidence that flies are capable of successfully fending off an EPN infection (See figure S1. Each line represents at least 90 flies). So, while the fly is a powerful model of immunity, and we have used it to develop a powerful model for studying immune modulation, the fruit fly is not a good host for EPN infections. Therefore, we proceeded with a bacterial infection model to assess FAR’s effects on immunity and infection.

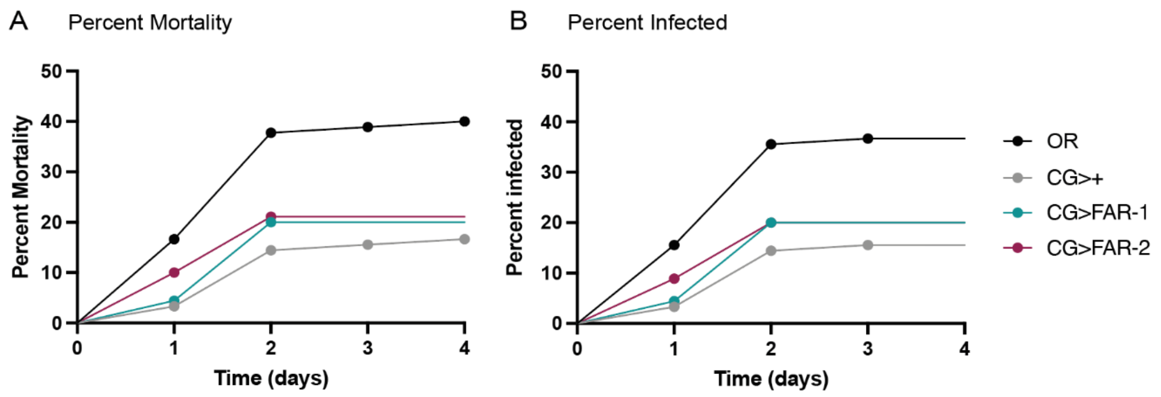

Figure S1

The second obstacle is that Sc releases hundreds of proteins during active infection, and many of these are predicted to alter lipid abundance. For example, there are several phospholipase A2 enzymes released by Sc during active infection, these cleave fatty acids from the cell membrane. There are also 3 other FARs released by Sc that we have not included in this study that may also affect lipid abundance during infection. In addition, the nematodes release pathogenic bacteria. For Sc this bacterium is *Xenorhabdus nematophila*, and this bacterium is known to produce and release its own arsenal of immunomodulatory effectors, including an inhibitor of endogenous insect PLA2 (Park et al., 2004). For these reasons we do not think that monitoring the lipid composition during a natural Sc infection would be helpful; the additional variables in that experiment make it incomparable to the FAR and control experiments we have performed.

Griffin, C.T. 2012. Perspectives on the behavior of entomopathogenic nematodes from dispersal to reproduction: Traits contributing to nematode fitness and biocontrol efficacy. Journal of Nematology 44(2):177-184.

Park Y., Kim Y., Tunaz H., and Stanley D.W. 2004. An entomopathogenic bacterium, Xenorhabdus nematophila, inhibits hemocytic phospholipase A2 (PLA2) in tobacco hornworms Manduca sexta. Journal of Invertebrate Pathology 86(3):65-71.

Iberkleid, I., Vieira, P., de Almeida Engler, J., Firester, K., Spiegel, Y., and Horowitz, S.B. (2013). Fatty acid-and retinol-binding protein, Mj-FAR-1 induces tomato host susceptibility to root-knot nematodes. PLoS One *8*, e64586.

3) The observation that FARs bind certain FAs is clearly shown, but whether this functionally linked to their immunesuppressive effect remains speculative. Therefore the authors should take advantage of their expression systems to generate FAR-variants in which putative FA-binding domains are mutated. This mutant FARs should then be tested for their ability to suppress immune responses and compromise bacterial clearance in the flies.

We agree with the reviewer that determining that the effect of FAR is a result of binding, and then determining which binding pocket, the fatty acid pocket or the retinol binding pocket, is needed for this effect would enhance our mechanistic understanding of how FAR suppresses immunity. However, we believe that the amount of work required to do this makes this a great set of experiments for a follow-up study.

Due to the high sequence diversity of known FARs, the exact fatty acid binding residues were difficult to identify. There is a crystal structure for only one FAR protein, FAR-7 from C. elegans (Jordanova et al., 2009). We could identify the putative binding pockets of FAR-1 and FAR-2 based on homology with FAR-7. Abolishing the binding ability of either pocket would require extensive trial and error and would need to be done using recombinant proteins first, before doing this in a transgenic fly, to verify that the binding activity had been abolished. There is both a fatty acid- and a retinol-binding pocket, so each pocket would need to be mutated independently, and then a mutant with both pockets mutated would need to be made as well. Binding affinity of these mutants would then need to be evaluated *in vitro* to confirm that the mutations used affected the binding. We think this would make an excellent follow up paper, but it will require an enormous amount of work and is beyond the scope of the current study.

Minor Issues:
1) Are there no significant differences in figure 1C?

The statistics done here are an unpaired t-test and although a clear trend is observed, there is no significant difference. The data is plotted on a logarithmic scale for easy visualization however there are a lot of overlapping data points between the groups yielding an insignificant change.

2) they authors should better discuss the similarities or differences in function between FAR1 and 2. Would there be an additive effects of FAR1 and 2 are administered simultaniously?

We performed the additive recombinant FAR injection experiments and found no difference between a 250 ng dose of FAR-1 or FAR-2 and a 125 ng FAR-1 plus 125 ng FAR-2 combined dose showing that FAR does not have an additive effect. FAR-1 and 2 seem to have similar *in vivo* effects at least at the level of the outcome of infection, leading us to believe that downstream immune effects are also being affected in similar ways not specific enough to evaluate their differences. The graph below shows at least 180 flies per line with statistical significance shown as a log-rank test.

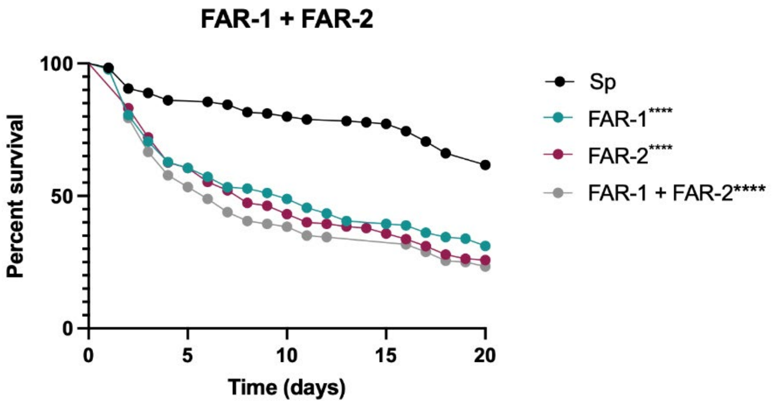

Reviewer 3:
The current study by Parks et al is an exciting study on Fatty acid and retinol binding proteins in a parasitic nematode species of insects, S. carpocapsae. The central idea is that FARs are

widely expressed across multiple species and these proteins are found within E/S products that would impact host biology. Indeed the authors show that either by recombinant protein injection of FAR's or transgenic overexpression in Drosophila, that FARS impair the response to bacterial challenge and lead to reduced host survival, which. is correlated with reduced phenoloxidase activitiy, melanization and AMP activity. They go on to demonstrate binding activity of FARS and show that at least for FAR-1, that over-expression. in teh. fly reduces metabolite abundance in the hemolymph. This is a comprehensive and potentially highly impact. ful study

Major Issues:
Most of my issues have to do with completeness of the data sets and issues with data display and interpretation.

1. FIg 1B, it seems like 20 and 50ng recombinant FAR-2 enhances survival which is not discussed at all and is contrary to the main hypothesis. Is this data consistent? The authors need to make an interpretation

We appreciate this concern, and we think it is one of the most interesting aspects of our findings. We speculate that this improvement in survival is due to the suppression of immune-induced damage. Phenoloxidase activity and subsequent melanization can be harmful to the fly. If FAR suppresses the immune system enough to prevent immune-induced damage but not enough to reduce immune resistance, this would lead to the phenotype we observe in figure 1B. We did address this in our discussion section on page 15. “During a bacterial infection the host is harmed by two factors: the pathogen and the immune response. In experiments with FAR-2, low doses (20 ng and 50 ng) led to significantly improved survival, which we hypothesize is due to the suppression of immune-induced damage.”

2. There are no experiments that use a transgenic over-expression of an irrelevant protein instead of just empty vector as control.

We appreciate this concern and recognize that this is a common control in mammalian systems. We have now performed such an experiment where we expressed mCherry using the CG driver. In addition to this new experiment, our other presented experiments include several controls that address this concern, and there are some data points that address it as well:
A) We injected heat-killed recombinant FAR, for both Sc-FAR-1 and Sc-FAR-2, 250 ng (S-Fig 4). We found that injecting the heat-killed FAR had no effect when co-injected with Streptococcus pneumoniae.
B) We performed lifespan experiments on FAR-expressing flies (S-Fig 8) and showed that there is no effect on the lifespan of Sc-FAR-1-expressing flies, and only a minor effect on Sc-FAR-2-expressing flies, that manifests itself after ~30 days of life.
C) In Figure 1B, we found that 100 ng and 200 ng of Sc-FAR-2 had no effect on fly survival.
D) We find concordance of the overexpression data and the recombinant injection data.
E) The recombinant protein coinjections have different phenotypes (Figure 1), suggesting that the it is the active FAR protein leading to the phenotypes.

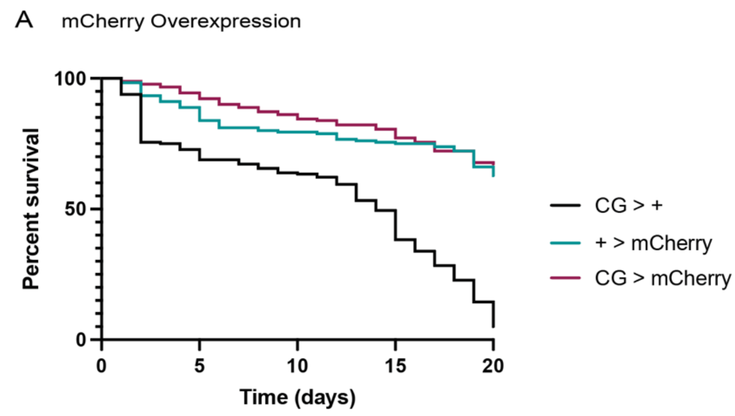

When mCherry is overexpressed by the CG-Gal4 driver, there is no significant reduction in survival. Here we point out that there is no difference in survival between expression of mCherry and its cross to wild-type OregonR. However, there is a difference between flies with varying genetic backgrounds, where the mCherry parent background is more susceptible to an *S.p* infection.

3. FIg. 4 is a critical part of this work. Why is only FAR1 expressing flies shown. This definitely needs to include FAR2 because there was different efficacy between the two genes.

We appreciate this comment and we have gone back and redone this entire experiment, including both FAR-1- and FAR-2-expressing flies, as requested. We redid the entire metabolomics analysis rather than just performing the FAR-2 experiment to guard against uncontrolled variability such as variation in batches of food, weather-associated variations in humidity, etc. As it turns out, the newly generated data lead to the same conclusion as the previous experiment. We can now draw these conclusions for both FAR-1 and FAR-2.

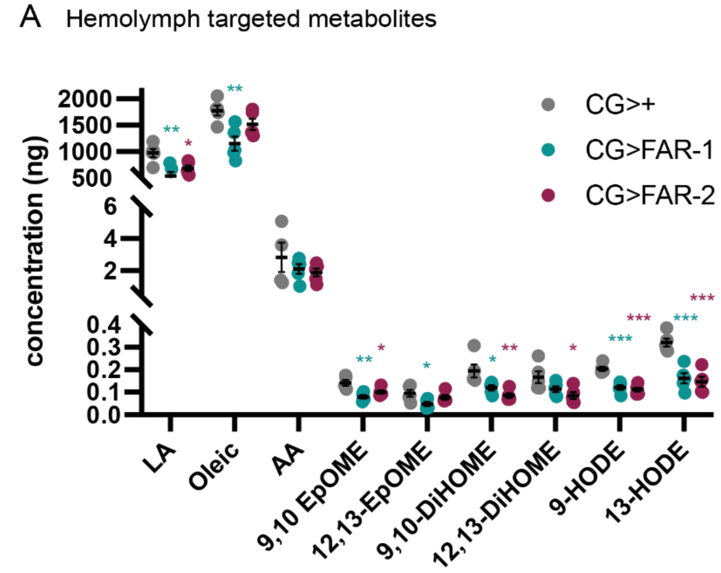

<u>Minor issues:</u>

4. Gene alignment data should be included for the FAR genes across species.

We appreciate this comment and agree that gene alignment can be useful. A phylum-level analysis investigating 576 FAR proteins from 58 nematode was recently published by another research group (Yuan et al., 2021). That was a much more comprehensive study of sequence evolution than was appropriate in this manuscript, and we have cited it in our manuscript. We thought that with the extensive data already included in this manuscript, 6 figures in the main text and 15 supplemental figures, that a gene alignment was unnecessary.

Yuan, D., Li, S., Shang, Z. *et al.* Genus-level evolutionary relationships of FAR proteins reflect the diversity of lifestyles of free-living and parasitic nematodes. *BMC Biol* **19,** 178 (2021). https://doi.org/10.1186/s12915-021-01111-3

5. Why is L. monocytogenes used for the data panels in Fig. 3,?

L. monocytogenes is much better at eliciting a robust melanization response. We have mentioned this in the results section on page 13.
"Disseminated melanization and PO activity was measured post-injection with *L. monocytogenes*, which elicits a robust disseminated melanization phenotype 4 days post injected which elicits a robust disseminated melanization phenotype 4 days post injected."

Also, previous work (Ayres) identified pathogens as melanizers and non-melanizers and using a melanizing pathogen is necessary to measure outputs of that cascade.

Ayres, J.S., and Schneider, D.S. (2008). A signaling protease required for melanization in Drosophila affects resistance and tolerance of infections. PLoS Biol *6*, 2764-2773.

6. Throughout the manuscript, the labelling of the figure legend is confusing and is difficult to discern, they should be reordered from left to right in ascending concentration

We appreciate this comment but feel that our figures are very clearly labeled with clear color coordination and shape patterning. We do have our figure legends labeled from left to right and in ascending concentration.
